# Bone metabolism in patients with type 1 neurofibromatosis: key role of sun exposure and physical activity

**DOI:** 10.1038/s41598-022-07855-4

**Published:** 2022-03-14

**Authors:** Ursula Pia Ferrara, Cristina Tortora, Carmen Rosano, Antonia Assunto, Alessandro Rossi, Stefano Pagano, Mariateresa Falco, Chiara Simeoli, Rosario Ferrigno, Alessandra D’Amico, Dario Di Salvio, Giuliana Cangemi, Rosario Pivonello, Pietro Strisciuglio, Daniela Melis

**Affiliations:** 1 Department of Translational Medical Science, Section of Pediatrics, Federico II University, Naples, Italy; 2 Department of Molecular Medicine and Medical Biotechnology, Federico II University, Naples, Italy; 3 Pediatric Unit, San Giovanni di Dio e Ruggi D’Aragona University Hospital, Salerno, Italy; 4 Department of Clinical Medicine and Surgery, Endocrinology Section, Federico II University, Naples, Italy; 5 Radiology Department, “Federico II” University, Naples, Italy; 6 Clinical Pathology Laboratory, Istituto Giannina Gaslini, Genoa, Italy; 7 Department of Medicine, Surgery and Dentistry, Scuola Medica Salernitana, University of Salerno, Fisciano, Italy

**Keywords:** Clinical genetics, Medical genetics

## Abstract

Bone metabolism has been rarely investigated in children affected by Neurofibromatosis type 1 (NF1). Aim of the present study was to assess bone mineral metabolism in children and adults NF1 patients, to determine the relevant factors potentially involved in the development of reduced bone mineral density (BMD), and provide possible therapeutic intervention in NF1 patients. 114 NF1 patients and sex and age matched controls were enrolled into the study. Clinical and biochemical factors reflecting bone metabolism were evaluated. Factors potentially affecting BMD were also investigated including: physical activity, sun exposure, vitamin D intake. Whenever the presence of vitamin D deficiency was recorded, cholecalciferol supplementation was started and z-score data obtained at Dual-Energy X-ray Absorptiometry (DXA) during supplementation were compared with previous ones. NF1 patients showed lower Z-scores at Dual-Energy X-ray Absorptiometry DXA than controls. Physical activity was significantly reduced in NF1 patients than in controls. Sun exposure was significantly lower in NF1 compared to control subjects. At linear regression analysis vitamin D was the most predictive factor of reduced z-score at DXA (p = 0.0001). Cholecalciferol supplementation significantly increased BMD z-score (p < 0.001). We speculated that a combination of different factors, including reduced sun exposure, possibly associated with reduced serum vitamin D levels, and poor physical activity, concur to the impaired bone status in NF1 patients. We also demonstrated that treatment with vitamin D can be effective in improving z-score value in NF1 patients, including children. In conclusion, the findings of the current study are expected to have important implications for the follow-up and prevention of osteopenia/osteoporosis in this common genetic disease.

## Introduction

Neurofibromatosis type 1 (NF1, OMIM #162200) is one of the most common autosomal dominant disorders with multisystem involvement; affects approximately 1/3500 live births^1^; it is characterized by inter- and intra-familial clinical variability. Major features include cafe-au-lait macules (CALMs), skinfold freckling (SF), Lisch nodules (LN), neurofibromas (NF), typical bone abnormalities, and optic pathway glioma (OPG)^1^. Diagnosis is based on the criteria defined at the National Institute of Health 1988 NF consensus conference^2^.

NF1 is caused by heterozygous mutations of the *NF1* gene (chromosome 17q11.2) which contains 57 constitutive and 3 alternatively spliced exons^3^ and encodes neurofibromin, a protein with tumor suppressor function, ubiquitously expressed and involved in pathways affecting cell growth and development of skeletal, cardiovascular, and nervous systems^4^. Neurofibromin is a member of the GTPase-activating protein gene family and can modulate adenylyl cyclase and protein kinase A (PKA), regulators of osteoblasts and osteoclasts cell function. Decreased expression of neurofibromin is correlated with dose-dependent elevation of intracellular RAS-activity and increasing expression of intracellular signaling pathways (mitogen-activated protein kinase, phosphatidil inositol-3-phosphate kinase), and decreasing expression of c-fos, a crucial regulator in osteoblast differentiation^5^.

The large majority (90–95%) of disease-causing *NF1* mutations includes intragenic mutations, and less than 10% is represented by large deletions encompassing the entire *NF1* gene and its flanking genomic regions at 17q11.2^6–11^. Most intragenic mutations are truncating or intragenic copy number changes, a smaller percentage are “in frame” variants^12^.

As bone features are concerned, NF1 patients are shorter than healthy subjects: several studies highlight proportionated short stature between 8 and 15% of patients affected by NF1, thus suggesting a generalized skeleton bone growth decrease^13–16^. Previous studies, performed in NF1 patients, showed local and general dysregulation in bone resorption and remodeling^14,17,18^, and increased formation of osteoclasts^19^. An increased bone fracture rate was observed in NF1 adults^20^ and in NF1 postmenopausal women^21^. Reduced bone mineral density (BMD), osteoporosis and increased fracture risk were also found in young NF1 patients^22^. Previous studies reported low levels of serum 25-hydroxy vitamin D3 (25OHD) and reduced BMD and trabecular bone density in both adults and children NF1 patients. Although skeletal involvement in patients with NF1 is well known, and osteoporosis has been reported in adult NF1 patients, there is limited knowledge about bone metabolism in NF1 children^13–19^.

The aim of the present study was to assess bone mineral metabolism in children and adults NF1 patients, to determine the relevant factors potentially involved in the development of reduced BMD, and provide possible therapeutic intervention.

## Patients and methods

### Patients and controls

Patients affected by NF1 and followed at the Department of Translational Medicine, Federico II University of Naples, Pediatric Section, were enrolled in the study after the protocol was discussed with each patient (or legal tutor) and informed consent was obtained. Patients’ clinical data were obtained from medical records over the past 20 years.

A multidisciplinary team, including endocrinologists, geneticists and radiologists, evaluated all patients.

The enrollment was carried out according to the following inclusion criteria: (i) clinical diagnosis of NF1 based on recommendations of National Institute of Health^2^; (ii) informed consent expression. The exclusion criteria were: (i) concurrent disorders affecting bone metabolism, i.e., primary hyperparathyroidism, hyperthyroidism, renal or hepatic chronic disease, malabsorption, hypercortisolism, hypogonadism; (ii) previous or ongoing treatments with glucocorticoids, chemotherapies, bisphosphonates or other antiosteoporotic drugs; (iii) previous spine or hip fractures; (iv) surgical correction of scoliosis.

Collected clinical information included familiarity, presence or absence of CALMs, intertriginous SF, LN, cutaneous and subcutaneous NFs, plexiform NFs, spinal NFs, bone abnormalities, OPGs, cardiovascular malformations, endocrine system involvement, developmental delay/intellectual disability, cerebrovascular malformations, and occurrence of other neoplasms.

Patients were divided into three groups according to the severity of the phenotype using the classification proposed by Riccardi^20^. Patients presenting with CALMs, axillary freckling, LN, dermal and/or nodular NFs, and non-progressive scoliosis were classified as “mild”, those presenting with plexiform NFs, bone abnormalities, precocious or progressive scoliosis were classified as “moderate”, and patients with learning disability/mental retardation, OPG and/or other neoplasms, and/or cerebrovascular disease were classified as “severe”.

114 individuals diagnosed with NF1 according to National Institutes of Health criteria^2^ were enrolled into the study. 69 patients were females and 45 males. The average age at time of study entry was 11.9 ± 5.6, range 1–23 years. 40 patients were children (aged between 1 and 11 years), 34 were in pubertal age (aged between 12 and 16) and 40 were adults (aged between 17 and 23).

Based on the phenotype, 31 patients were classified as “mild” (mean age 12.8 ± 6, range 2–22 years), 39 as “moderate” (mean age 12.3 ± 5.9, range 2–22 years), and 44 as “severe” (mean age 12.3 ± 5.5, range 1–23 years). Demographic and clinical characteristics are reported in Table 1.

**Table 1:** Demografic and clinical characteristics of the 114 patients with NF1 included in the study.

Feature	Mild phenotype (n = 31)	Moderate (n = 39)	Severe phenotype (n = 44)	Whole cohort (n = 114)
Mean age (average) ± SD	13 ± 6.0 years (2–22 years)	12 ± 6.9 years (2–22 years)	12.3 ± 5.9 years (1–23 years)	11.9 ± 5.6 years (1–23 years)
No mutation	N = 8	N = 8	N = 8	N = 24
Gender	M = 12; F = 19	M = 13; F = 26	M = 20; F = 24	M = 45; F = 69
CALMs	31 (100%)	39 (100%)	44 (100%)	114 (100%)
Lisch nodules	11 (35.4%)	20 (51. 3%)	25 (56.8%)	56 (49.1%)
Axillary and/or inguinal freckling	23 (74.2%)	33 (84.6%)	33 (75%)	89 (78%)
Plexiform neurofibroma	0 (0.0%)	6 (15.3%)	9 (20.4%)	15 (13.2%)
Mild non-progressive scoliosis	12 (38.7%)	18 (46.1%)	NA	4 40 (35.1%)
Progressive scoliosis	0 (0.0%)	14 (35.9%)	25 (56.8%)	39 (34.2%)
Heart involvement	4 (12.9%)	7 (17.9%)	12 (27.3%)	23 (20.2%)
OPG	0 (0.0%)	0 (0.0%)	27 (61.4%)	27 (23.6%)
Other tumors	0 (0.0%)	0 (0.0%)	17 (38.6%)	17 (14.9%)
Development delay and/or cognitive deficit	0 (0.0%)	0 (0.0%)	28 (63.6%)	28 (24.5%)

*F* females, *M* males, *NA* not available.

All patients were screened for *NF1* and *SPRED1* mutations by parallel sequencing of the whole coding region and flanking splice sites (± 10 bp). Structural rearrangements were assessed by Multiplex Ligation Probe Amplification analysis using the MRC-Holland P295 probe set. A pathogenic or likely pathogenic *NF1* variant was found in 90/114 (78.9%).

114 age- and sex-matched healthy controls were also enrolled. 69 were females. The average age at time of study entry was 12.5 ± 5 years (range 3–24 years). 40 were children (aged between 3 and 11 years), 34 were in pubertal age (aged between 12 and 16) and 40 were adults (aged between 17 and 23). Normal auxological parameters were recorded in the control group.

To minimize potential source of bias, none of the controls had disorders or treatments affecting bone metabolism or BMD.

To evaluate the efficacy of vitamin D therapy in patients with vitamin D insufficiency, 57 patients were followed for a mean period of 2,4 ± 0.9 years (range 2–5 years) after starting 25OH- D supplementation.

## Methods

This is a restrospective study recording data obtained during 20-year follow-up. All methods were carried out in accordance with relevant guidelines and regulations. All experimental protocols were approved by “Comitato Etico Università Federico II”, protocol number 315/18.

### Clinical evaluation

Short stature was defined as height less than two standard deviations. Pubertal stage was assessed according to Tanner's pubertal stages; growth velocity was also evaluated.

### Life style

In order to assess the potential contribution of “environmental” factors, physical activity, sun exposure, vitamin D and calcium intake were recorded during the enrolment visit.

Physical activity was evaluated by administering the International Physical Activity Questionnaire (IPAQ)^21^. For each patient the time spent experiencing different kind of activities during the previous week was calculated: vigorous-intensity activity (hard physical effort and the patient breathe harder than normal), moderate-intensity activity (moderate physical effort, walking) and the time spent sitting.

Sun exposure was considered low with at least two of the following criteria: no arm and skin exposure during summer months; no sunbathing or holiday in sunny places; no working outdoors. Otherwise, it was deemed sufficient^22,23^.

To quantify the dietary intake of calcium and vitamin D a FFQ (food frequency questionnaire) was administered to the patients and controls; we asked them if they assumed calcium-rich foods/vitamin D-rich foods and how often they did it, so we were able to quantify the amount of their daily calcium and vitamin D intake^24,25^.

Diet calcium intake was defined low with at least two of the following criteria: milk assumption less than 100 mL a day; less than three yogurts in a week; eating cheese less than two times in a week. Otherwise, it was deemed sufficient^24^.

Diet vitamin D intake was evaluated through a questionnaire evaluating intake of vitamin D-rich-food as portion per week. These specific food included vitamin D enriched cereals, cheese, fish, lentils. Diet Vitamin D intake was defined low with less than 3 portions assumed in a week. Otherwise it was deemed sufficient^24^.

### Biochemical markers of bone metabolism

Bone metabolism was studied evaluating serum calcium, phosphorus, alkaline phosphatase, parathyroid hormone (PTH), calcitonin, 25OHD, C-terminal telopeptide of type I collagen (CTX) and osteocalcin (OC) and urinary calcium/creatinine ratio (UCa/UCr). CTX was measured using a serum cross-laps enzyme-linked immunosorbent assay (ELISA) kit (Immunodiagnosticsystems, Frankfurt, Germany) and OC was measured using a Microvue Osteocalcin ELISA kit (Quidel corporation, San Diego, CA, USA). ELISA tests were automated on a DSX system (Dynex technologies, Technogenetics, Milan, Italy) following the manufacturer’s instructions. The other markers of bone metabolism were measured using immunoassay with commercially available kits.

Considering the vitamin D levels, NF1 patients were classified according to the Endocrine Society’s latest guidelines for vitamin D levels^26^: deficiency was defined by 25OHD less than or equal to 20 ng/ml; insufficiency when it was between 21 and 29 mg/ml; normal if it was more than 30 ng/ml.

Whenever the presence of vitamin D deficiency was recorded, vitamin D supplementation was started and BMD data obtained during vitamin supplementation were compared with previous BMD data. Cholecalciferol was used at a dose of 2,000 UI /day in prepubertal patients and 4,000 UI/day in adolescent^27^.

### Hormonal studies

To address the impact of endocrine regulation on bone homeostasis, the different endocrine axes were evaluated. The somatotropic axis was evaluated by analyzing basal serum growth hormone (GH) and insulin-like growth factor 1 (IGF-1) via immune assay (CLIA); the thyrotropic axis function by analyzing serum thyroid-stimulating hormone (TSH), free triiodothyronine (fT3), free thyroxine (fT4), T3, T4; the adrenocorticotropic axis function by analyzing plasma adrenocorticotropic hormone (ACTH), serum cortisol, androstenedione, 17hydroxyprogesteron (17OHP), dehydroepiandrosterone sulphate (DHEA-S), renin, aldosterone levels and 24-h urinary free cortisol (UFC); the gonadotropic axis function by analyzing serum follicle-stimulating hormone (FSH), luteinizing hormone (LH), 17β-estradiol, testosterone levels. The beta-cell function was analyzed by evaluating basal serum insulin levels, all measured by using immunoassay with commercially available kits.

### Bone mineral density

BMD was studied using Dual-emission X-ray absorptiometry (DXA). DXA (Hologic QDR 1000, Hologic Inc., Waltham, USA). Osteopenia and osteoporosis are commonly diagnosed in men over 50 and postmenopausal women according to bone health guidelines using the T-score. In younger individuals, the Z-score is used. This parameter only has a cut-off point of − 2DS, in this case, the individual has a bone mass below that expected for their age^28,29^.

Since both children and adult participants were enrolled, BMD was measured at the L1–L4 vertebrae, considering that the hip is not a reliable site for measurement in growing children. Z-scores were calculated by comparing BMD with age and sex matched reference values according to the manufacturer’s internal reference database.

### Statistical analysis

All data mentioned in the text or shown in the figure are expressed as mean ± standard deviation (SD).

Statistical analysis was performed using Statistical Package for Social Science (SPSS 18) for Windows Update; SPSS Inc., Chicago, Illinois, USA). The comparisons between numerical variables were performed by Student’s t test corrected for Fisher’s exact test. Pearson’s correlation test was performed to assess the relationship among variables with normal distribution (p < 0.05) was considered as statistically significant) whereas Spearman’s correlation test was performed to assess the relationship among variables with skewed distribution (a ρ < 0.005 was considered statistically significant). Logistic regression analysis was performed to determine independent predictors of patient outcome (between bone mineral marker and z-score). Univariate analysis of variance and covariance analysis were performed to eliminate the effects of age and gender.

## Results

The number of patients at each stage of the study is shown in Additional File 1. At each stage patients were excluded either due to data unavailability (bone metabolism study) or unwillingness to start vitamin D treatment (vitamin D follow-up study).

### Clinical evaluation

Short stature was detected in 13/114 patients (11.4%), particularly in 2/31 (6.45%) with mild phenotype, 3/39 (7.69%) with moderate phenotype and 8/44 (18.18%) with severe phenotype. Precocious puberty was detected in two patients with severe phenotype (4.54%).

### Life style

Physical activity was significantly reduced in NF1 patients than in controls. Particularly both vigorous-intensity activity (0.24 ± 0.2 h vs 10.5 ± 3, p < 0.001) (Table 2), moderate-intensity activity (2.81 ± 0.2 vs 6.98 ± 2.5, p = 0.012) and walking (3.09 ± 0.2 vs 7.2 ± 2.5, p = 0.02) (Table 2) appeared significantly reduced. The time spent sitting every day was higher in patients than in controls (51 ± 8 vs 32.8 ± 7, p = 0.01) (Table 2).

**Table 2:** Life style results in patients and controls.

	Patients (n = 114)	Controls (n = 114)	p-value
Vigorous-intensity activity (h/day) M ± SD	0.24 ± 0.2	10.5 ± 3	p < 0.001
Moderate-intensity activity (h/day) M ± SD	2.81 ± 0.2	6.98 ± 2.5	0.012
Walking (h/day) M ± SD	3.09 ± 0.2	7.2 ± 2.5	0.02
Time spent sitting (h/week) M ± SD	51 ± 8	32.8 ± 7	0.01
Low sun exposure (%)	59.6	21.9	p < 0.001
Low diet calcium intake (%)	46	55	0.18
Low vitamin D intake (%)	69	66	0.12

Sun exposure was low in 68/114 (59.6%) patients and in 25/114 (21.9%) control subjects. Overall, sun exposure was significantly lower in NF1 compared to control subjects (Chi square: 33.8; p: 0.00; OR 5.26) (Table 2).

Diet calcium intake was low in 46% and in 55% of patients and the control group, respectively. No significant difference was observed between patients and controls (Chi square: 0.18; p: 0.6733; OR 0.7) (Table 2). Vitamin D intake was low in 69% and in 66% of patients and the control group, respectively. No significant difference was observed between patients and controls (Chi square: 0.12; p: 0.7279; OR 1.38) (Table 2).

### Biochemical markers of bone metabolism

Biochemical parameters were evaluated in 108 patients (28 with mild phenotype, 36 with moderate phenotype and 44 with severe phenotype). NF1 patients showed decreased levels of calcium, calcitonin and 25OHD, and increased levels of both OC and CTX compared to controls (Table 3). Calcitonin serum levels were low in 51/108, 47.2% of the total group of patients, 18/28 (64.2%) with mild phenotype, 14/36 (38.8%) with moderate phenotype, 19/44 (44.7%) with severe phenotype. Vitamin D serum levels were low in 57/108 patients, 52.7% of the total group of patients, 10/28 (35.7%) with mild phenotype, 18/36 (56.25%) with moderate phenotype, 29/44 (66.6%) with severe phenotype.

**Table 3:** Biochemical markers of bone metabolism and bone mineral density in patients and controls.

	Patients	Controls	p
**Phenotype**			
Calcium mg/dl	9.6 ± 0.88	9.9 ± 0.4	0.009
Calcitonin pg/ml	3.4 ± 2.7	9.6 ± 2	p < 0.01
Osteocalcin ng/ml	121 ± 15	67 ± 5	p < 0.01
CTX ng/ml	1.64 ± 0.04	0.42 ± 0.005	p < 0.01
Vitamin D ng/ml	21 ± 7.3	45 ± 15	p < 0.01
z-score	− 1.1 ± 1	0.1 ± 0.9	p < 0.01
**Mild**			
Calcitonin pg/ml	3.5 ± 2	9.2 ± 1.4	p < 0.01
Vitamin D ng/ml	19.5 ± 7	44.3 ± 12	p < 0.01
z-score	− 1 ± 0.8	0.2 ± 0.9	0.02
**Moderate**			
Calcitonin pg/ml	3.15 ± 2.3	9.3 ± 2.1	p < 0.01
Vitamin D ng/ml	25.6 ± 17	47.4 ± 15	p < 0.01
z-score	− 0.9 ± 1.2	0.04 ± 0.9	0.0005
**Severe**			
Calcitonin pg/ml	4.8 ± 3	10 ± 2	p < 0.01
Vitamin D ng/ml	15.2 ± 6	43.9 ± 15	p < 0.01
z-score	− 0.6 ± 1.4	0.16 ± 0.9	0.01

### Hormonal studies

No significant differences were observed between patients and controls.

### Bone mineral density

DXA was performed and z-score was evaluated in 108 patients (28 with mild phenotype, 36 with moderate phenotype and 44 with severe phenotype).

NF1 patients showed lower Z-scores at DXA than controls (Table 3).

BMD value below age reference were detected in 32/108 patients (29.6%); particularly in 4/28 (14.2%) with mild phenotype, 9/36 (25%) with moderate phenotype and 19/44 (43.18%) with severe phenotype.

### Correlation studies

The DXA Z-scores correlated directly with 25OHD serum levels (r = 0.533, p = 0.0001); 25OHD serum levels also inversely correlated with PTH serum levels (r = − 0.253, p = 0.002), ALP (r = − 0.387, p = 0.001) and directly with calcium serum levels (r = 0.18, p = 0.01).

Moderate-intense physical activity correlated with both alkaline phosphatase (r = 0.25, p = 0.007) and PTH serum levels (r = 0.19, p = 0.004); moderate activity correlated with PTH serum levels (r = 0.65, p = 0.005); the time spent sitting every day correlated with calcitonin (r = 0.55, p = 0.018) and inversely correlated with PTH serum levels (r = − 0.638, p = 0.008).

**Figure 1:**
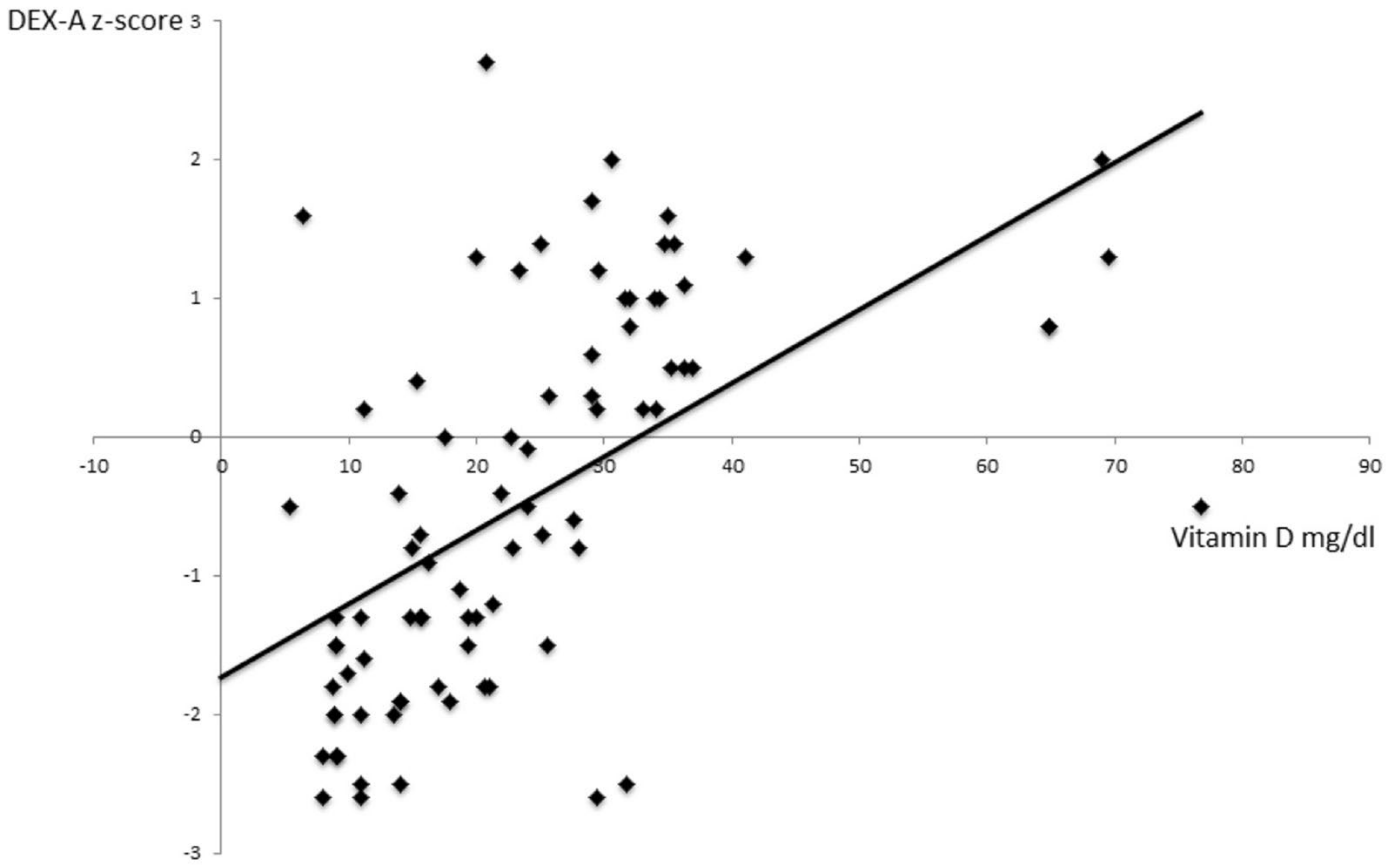
Correlation between vitamin D serum levels and bone mineral density values.

At linear regression analysis vitamin D was the most predictive factor of reduced z-score (beta = 0.45, p = 0.0001). (Fig. 1).

### Data obtained after at least 2 years of vitamin D supplementation

**Figure 2:**
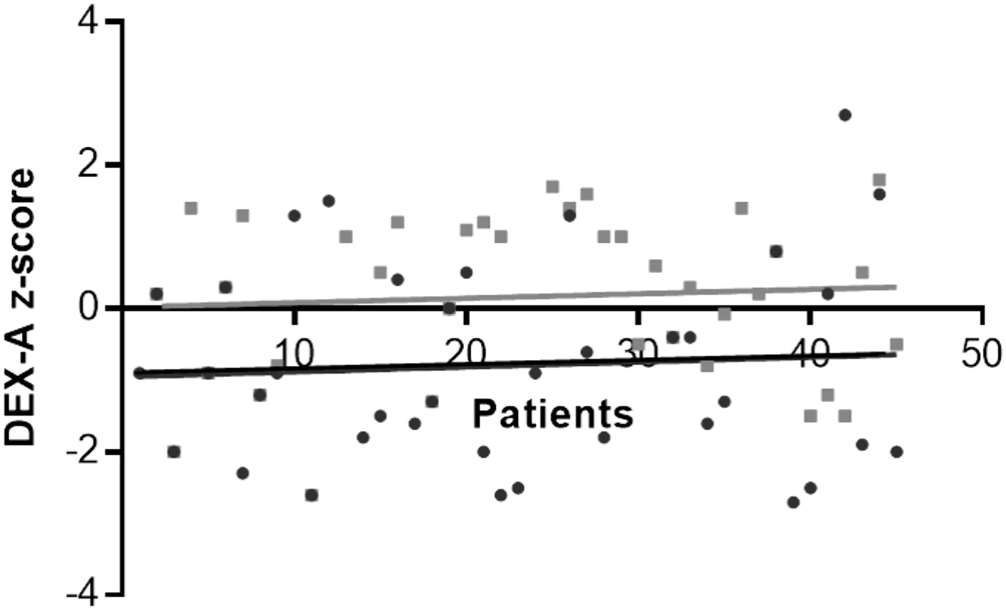
Bone mineral density data in patients at baseline (black) and after at least two years of cholecalciferol supplementation (grey).

Fifty-seven patients diagnosed with vitamin D insufficiency were followed for a mean period of 2.4 ± 0.9 years, range 2–5 years after starting cholecalciferol supplementation. Z-score after supplementation was significantly higher compared to baseline in NF1 patients (0.7 ± 1 vs − 1.1 ± 1, p = 0.000) (Fig. 2). No patients showed osteoporosis after two-years of vitamin D supplementation. Vitamin D serum levels significantly increased after supplementation (24 ± 12 vs 20 ± 12, p = 0.001), Alkaline phosphatase significantly decreased (175 ± 76 vs 159 ± 76, p = 0.04). Calcium (10.2 ± 8.8 vs 9.4 ± 1.2, p = 0.39), phosphorus (5.06 ± 5.4 vs 4.8 ± 4.3, p = 0.68) and calcitonin (3.3 ± 2.7 vs 3 ± 2.8, p = 0.35) serum levels were similar in the two groups of patients.

## Discussion

This study confirmed the presence of low BMD in NF1 patients, including children. Although osteopenia/osteoporosis have been reported in NF1, pathophysiology and correlations with underlying genetic defect are poorly understood^30–34^. To gather information on the factors that may be implicated, bone metabolism, physical activity, sun exposure, calcium and vitamin D intake, and hormonal status were assessed in a large cohort of 114 NF1 patients.

NF1 patients showed reduced calcium, calcitonin and vitamin D and increased OC and CTX serum levels when compared with age- and sex-matched healthy subjects. The observation of decreased levels of calcitonin, marker of osteoblastic activity and bone neo-formation in NF1 patients, is consistent with a previous report^35^. This finding might be considered as a compensatory mechanism against the increased resorption, which is in turn pointed out by high levels of CTX observed in NF1 patients.

The finding of reduced calcitonin and increased OC and CTX levels suggests the presence of both reduced bone deposition and increased bone remodelling. Impaired bone mineralization in NF1 patients may result from several factors^36^.

Reduced z-score at DXA might be due to negative effects of neurofibromin mutations on bone growth and structure^13,18^. The neurofibromin protein, independent of its Ras GTPase-activating activity, can modulate adenyl cyclase activity and PKA^3,11^. Since cAMP and PKA are primary signalling pathways regulating osteoblast and osteoclast cell functions in response to PTH, it is plausible that haploinsufficiency of *NF1* in humans can result in both decreased bone formation and/or increased osteoclastic activity and the clinical picture of adynamic bone^34^.

At the cellular level, osteoblasts showed an increase in proliferation and a decreased ability to differentiate and mineralize in vitro^33^. In addition to an osteoblastic defect, NF1(+/−) mice were found to contain elevated numbers of osteoclasts with increased survival, proliferation, migration, adhesion and resorptive activity^35^.

Reduced mineralization^12–26^ as well as hypovitaminosis D^36,37^ have been reported in NF1 patients; however the mechanism underlying the reduced 25OHD in NF1 is unknown. Noteworthy, both dietary calcium and vitamin D intake were normal in NF1 patients, without differences when compared with healthy subjects.

Indeed, the current study demonstrated both reduced vitamin D serum levels in NF1 patients and a significant correlation between vitamin D serum levels and DXA results. One possible interpretation is that patients with major skin involvement might avoid sun exposure because of aesthetic embarrassment or discomfort (e.g. high number of neurofibromas may impact clothing choice and decrease outdoor activity levels)^38–40^. Indeed, NF1 patients reported a significant reduction in sun exposure compared to control subjects in the current study. Nonetheless, in vitro studies on skin samples might investigate the presence of a derangement of vitamin D production and metabolism in NF1 patients^41^.

Endocrine imbalance may also be involved in bone abnormalities. The anabolic effect of IGF-1 on the bone is well known, regulating bone growth and enhancing osteoblast proliferation^42^. Recently it has been shown that IGF-1 promotes osteoblastic activity activating mTOR-pathway^43,44^. IGF-1 affects bone status also acting in a paracrine way, in response to mechanical load, such as during physical activity. An increased IGF-1 expression in osteocytes and osteoblasts in response to mechanical load^45,46^ has been reported. Conversely, the absence of mechanical *stimuli* decreases IGF-1-mediated signalling. This strict connection between bone and muscle is synthesized in “mechanostat theory”^47,48^. According to this model, muscle and bone respond concertedly to each other's modification and to external factors, creating a “Muscle-Bone Unit”. The central element of this model is the regulatory feedback loop between tissue strain and bone architecture^47,48^. On the basis of these considerations, the reduced physical activity might also contribute to reduced z-score value at DXA in NF1. It is noteworthy that the results of the current study demonstrated a significantly reduced physical activity in patients when compared to controls. The detection of a correlation between physical activity and both alkaline phosphatase and PTH serum levels suggest the role of physical activity on bone metabolism.

Although it has been demonstrated that nutrient intake plays an important role in maintaining bone health, the current study failed to demonstrate a reduced calcium and or vitamin D intake or alternatively a correlation between these nutrient’s intake and reduced z-score.

This is a retrospective study and this may represent the main study limit, other limits include the methods to evaluate nutrition intake and the lack of comparison of auxological data between control and NF1.

Based on the results of the current study, it is likely that a combination of different factors, including reduced sun exposure, possibly associated with reduced serum vitamin D levels, and poor physical activity, concur to the impaired bone status in NF1 patients.

In addition, this study demonstrated clearly that treatment with vitamin D can be effective in improving DXA data in NF1 patients, including children. Further studies are necessary to investigate the effects of other factors influencing these data in NF1 patients, to further delineate the optimal treatment strategy, and to determine the efficacy and safety of high-dose cholecalciferol in NF1children. Prospective clinical trials to determine whether more aggressive interventions such as bisphosphonates will translate into increased bone mass in NF1 are required.

In conclusion, the findings of the current study are expected to have important implications for the follow-up and prevention of osteopenia/osteoporosis in this common genetic disease.

## Supplementary Information

Supplementary Information.

## Abbreviations

25OHD: 25-Hydroxy vitamin D3
BMD: Bone mineral density
CALMs: Cafe-au-lait macules
CTX: *C**-**terminal cross**-**linking* Telopeptide of type I *collagen*
DXA: Dual-energy X-ray absorptiometry
LN: Lisch nodules
NF: Neurofibromas
NF1: Neurofibromatosis type 1
OC: Osteocalcin
OPG: Optic pathway glioma
PKA: Protein kinase A
PTH: Parathyroid hormone
SF: Skinfold freckling
