# Supplementary material for: Bone metabolism in patients with type 1 neurofibromatosis: key role of sun exposure and physical activity
Source: Sci Rep. 2022 Mar 14;12:4368. doi: 10.1038/s41598-022-07855-4 (PMC8921306; doi:10.1038/s41598-022-07855-4)
Supplement: Supplementary file 1 — Supplementary Information. [file 41598_2022_7855_MOESM1_ESM.pptx]

## Slide 1
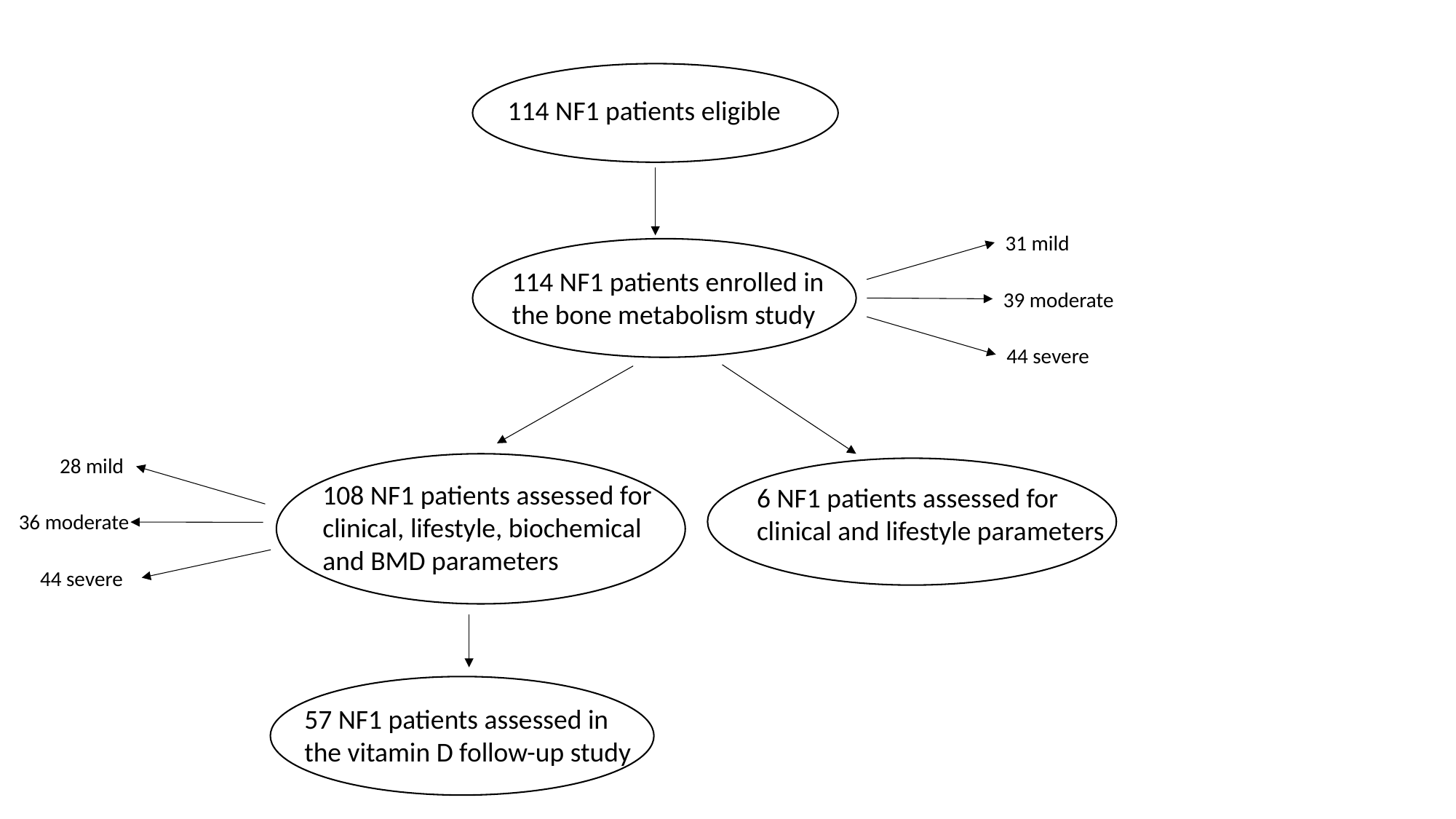

114 NF1 patients eligible
31 mild
114 NF1 patients enrolled in the bone metabolism study
39 moderate
44 severe
44 severe
36 moderate
28 mild
108 NF1 patients assessed for clinical, lifestyle, biochemical and BMD parameters
6 NF1 patients assessed for clinical and lifestyle parameters
57 NF1 patients assessed in the vitamin D follow-up study
